# A Scintillation Proximity Assay for Real-Time Kinetic Analysis of Chemokine–Chemokine Receptor Interactions

**DOI:** 10.3390/cells11081317

**Published:** 2022-04-13

**Authors:** Stefanie Alexandra Eberle, Martin Gustavsson

**Affiliations:** Department of Biomedical Sciences, Faculty of Health and Medical Sciences, University of Copenhagen, Blegdamsvej 3B, 2200 Copenhagen, Denmark; stefanie.eberle@sund.ku.dk

**Keywords:** 7TM receptor, ACKR3, CXCL12, SDF-1, chemokine, chemokine receptor, kinetics, association, dissociation, Scintillation Proximity Assay (SPA)

## Abstract

Chemokine receptors are extensively involved in a broad range of physiological and pathological processes, making them attractive drug targets. However, despite considerable efforts, there are very few approved drugs targeting this class of seven transmembrane domain receptors to date. In recent years, the importance of including binding kinetics in drug discovery campaigns was emphasized. Therefore, kinetic insight into chemokine–chemokine receptor interactions could help to address this issue. Moreover, it could additionally deepen our understanding of the selectivity and promiscuity of the chemokine–chemokine receptor network. Here, we describe the application, optimization and validation of a homogenous Scintillation Proximity Assay (SPA) for real-time kinetic profiling of chemokine–chemokine receptor interactions on the example of ACKR3 and CXCL12. The principle of the SPA is the detection of radioligand binding to receptors reconstituted into nanodiscs by scintillation light. No receptor modifications are required. The nanodiscs provide a native-like environment for receptors and allow for full control over bilayer composition and size. The continuous assay format enables the monitoring of binding reactions in real-time, and directly accounts for non-specific binding and potential artefacts. Minor adaptations additionally facilitate the determination of equilibrium binding metrics, making the assay a versatile tool for the study of receptor–ligand interactions.

## 1. Introduction

Approximately one third of the clinically approved drugs target seven transmembrane domain (7TM) receptors [1,2], which form the largest membrane protein family in the human genome with more than 800 members [3]. Strikingly, very few of these drugs (e.g., Mogamulizumab [4], Maraviroc [5] and Plerixafor [6]) target the over 20 human chemokine receptors, which belong to the class A 7TM receptors. The chemokine receptors and their endogenous ligands, the chemokines, contribute to a broad range of physiological processes such as cell migration, proliferation, development and homeostasis. Accordingly, the chemokine–chemokine receptor network is also extensively involved in various inflammatory and immune diseases including HIV and cancer [7,8,9], which makes them attractive drug targets. However, although new agents are currently in clinical trial, numerous others failed over the last 10–20 years due to the various challenges faced when developing drugs for chemokine receptors [10,11,12]. One such challenge is the complexity of the chemokine–chemokine receptor network. The network comprises conventional (cCKRs) and atypical chemokine receptors (ACKRs), and around 50 human chemokines [7,8]. Chemokine binding to cCKRs triggers intracellular signaling pathways, which, e.g., drives directed cell migration in response to chemokine gradients. ACKRs, in contrast to cCKRs, do not induce classical G protein-dependent signaling but can signal through G protein-independent pathways. Furthermore, ACKRs regulate the distribution, localization and abundance of chemokines, and thereby impede cCKR activation [13,14]. Several chemokines bind to multiple chemokine receptors and vice versa. In addition, chemokines and chemokine receptors can both form hetero- or homodimers, as well as higher-order oligomers, and undergo posttranslational modifications, all potentially altering their function [7,8]. Binding affinities, activated pathways and redundancy thereby depend on the specific receptor–ligand interaction and cellular context [7,15,16]. The promiscuity or redundancy of the chemokine–chemokine receptor network presumably evolved to increase the robustness of this system and consequently the protection against pathogens [7,16]. However, this inherent characteristic of the chemokine–chemokine receptor network entails special challenges in drug development [10,11,12,17]. Moreover, the binding interfaces between chemokines and chemokine receptors are relatively broad and flat [18], which complicates the design of ligands with highly specific binding and low off-target effects [10,19,20]. Other suggested reasons for clinical trial failures include poor drug-like properties or animal models. Regarding the latter, species differences, particularly of the immune and chemokine system, are emphasized [10,12,16,17].

One strategy to not only improve drug discovery but also obtain a deeper understanding of the complexity of the chemokine–chemokine receptor network is to include binding kinetic studies. Traditionally, receptor–ligand interactions were assessed by equilibrium binding metrics [21,22,23] such as the equilibrium dissociation constant (*K_d_*), equilibrium inhibition constant (*K_i_*), half-maximal inhibitory concentration (IC_50_) and the effector concentration for half-maximal response (EC_50_) [24]. However, these in vitro parameters of a closed system insufficiently represent the open, non-equilibrium system found in vivo [23,25]. Binding kinetics describe time-dependent changes in target engagement by the association (*k*_on_) and dissociation rate constant (*k*_off_). The *k*_off_ is independent of the local ligand concentration and describes the breakdown of a ligand–receptor complex [21,26]. The lifetime of the complex is given by the residence time (RT = 1/*k*_off_) [23,25]. RTs that are longer than the ligand elimination rate prolong the efficacy and allow a lower dosing [21,23,27,28]. This potentially reduces non-specific binding (NSB) and target-mediated toxicity, and can enhance the therapeutic window [21,23,27]. Moreover, long RTs provide a mechanism to deal with fluctuating local concentrations of endogenous ligand(s) by insurmountable antagonism [21,27,29]. However, long RTs bear the risk of inaccurate affinity measurements due to lack of equilibration [21,27,30,31,32]. Possible consequences in drug discovery include discontinued or unrealized hits, misinterpretation of target selectivity, wasted time and resources and safety issues due to overdosing [27,31]. Overall, optimal RTs depend on the specific situation. For example, fast but transient acting drugs, or cytotoxic radiopharmaceuticals require short RTs [23,27,33,34]. Other factors to consider are de novo target synthesis or target inactivation by degradation, desensitization or internalization [27,28,34,35]. The time course of the ligand–receptor complex formation is described by the observed association rate constant (*k*_on(obs)_). In contrast to *k*_off_, *k*_on(obs)_ depends on the local ligand concentration. Note that *k*_on(obs)_ and *k*_on_ are two distinct but related parameters (*k*_on(obs)_ = *k*_on_ [L] + *k*_off_) [27,30]. The *k*_on_ is linked to the onset of therapeutic action, as well as the duration and level of target occupancy [22,33]. In case of high proximal ligand concentrations, e.g., due to hindered diffusion, rebinding to the same or nearby targets can occur, extending the overall drug action [22,36,37,38].

In sum, binding kinetics provide a better prediction of in vivo efficacy than the equilibrium binding metrics [21,23,27,29,33]. Nevertheless, while the equilibrium binding metrics of the chemokine–chemokine receptor network are typically well characterized, less is known about its kinetic profile [39]. To address this issue, we present the application, optimization and validation of a Scintillation Proximity Assay (SPA) for real-time kinetic profiling of chemokine–chemokine receptor interactions on the example of the atypical chemokine receptor ACKR3 (previously known as CXCR7) and the chemokine CXCL12 (also known as SDF-1α). ACKR3 has two endogenous chemokines, CXCL12 and CXCL11 (also known as I-TAC), which also interact with the conventional chemokine receptors CXCR4 and CXCR3, respectively [40]. ACKR3 is considered an attractive drug target as, e.g., the CXCL12/CXCR4/ACKR3 axis is linked to various physiological and pathophysiological processes [41]. Based on the case study presented here on ACKR3, we propose the SPA as a powerful tool for kinetic profiling of the chemokine–chemokine receptor network. New insights into the binding kinetics of the chemokine–chemokine receptor system have the potential to provide a better understanding of its redundancy and specificity, and aid future drug-discovery campaigns.

## 2. Materials and Methods

### 2.1. Expression and Purification of ACKR3

Human ACKR3 (residues 2-362) expression and purification was performed as previously described [42], with slight modifications. Briefly, the ACKR3 gene (GenBank accession number NM_020311.3) was cloned into a pFastBac1 vector yielding an expression construct with a *C*-terminal FLAG and His_10_-tag, and an *N*-terminal GP64 promoter and hemagglutinin (HA) signal sequence. Recombinant baculovirus was generated using the Bac-to-Bac baculovirus expression system (Thermo Fisher Scientific, Roskilde, Denmark). *Spodoptera frugiperda* (*Sf9*) insect cells were infected with the baculovirus for protein expression. Cell membranes expressing ACKR3 were isolated using a Dounce homogenizer. ACKR3 was solubilized for 4 h in detergent containing buffer [0.75/0.15% (*w*/*v*) *n*-dodecyl-*β*-D-maltopyranoside (DDM)/cholesteryl hemisuccinate (CHS)] in the presence of ACKR3 agonist VUF11207 (Merck Life Science, Søborg, Denmark; [43]). Insoluble material was removed by centrifugation. The supernatant was supplemented with 20 mM imidazole and 1 unit/mL DNase I (Fisher Scientific, Roskilde, Denmark), and incubated with Talon IMAC resin (Takara Bio Europe, Saint-Germain-en-Laye, France) overnight. Resin was transferred to Poly-Prep chromatography columns (Bio-Rad, København, Denmark), washed with buffer (doubled HEPES pH 7.5 and imidazole concentrations compared to the original protocol) and the purified receptor was eluted with imidazole. The buffer of the eluted protein was exchanged to exchange buffer [50 mM HEPES pH 7.5, 400 mM NaCl, 0.025/0.005% (*w*/*v*) DDM/CHS, 10% (*v*/*v*) glycerol] by PD MiniTrap G-25 columns (Merck Life Science, Søborg, Denmark), and the protein subsequently concentrated as described in [44]. ACKR3 purity was assessed by SDS-PAGE.

### 2.2. Expression and Purification of CXCL12

Recombinant human CXCL12 was expressed and purified as previously described [42], with the exception that the time for protein expression was reduced to 4 h, and the storage buffer changed to 20 mM acetate pH 4. Briefly, His-tagged CXCL12 was expressed from a pET-21-based vector, with a preceding enterokinase cleavage site, in *Escherichia coli* BL21(DE3)pLysS (Agilent, Glostrup, Denmark). The His-tagged CXCL12 was affinity purified by means of Ni-NTA agarose resin (Qiagen, København, Denmark). His-tags were cleaved by enterokinase (Bionordika, Herlev, Denmark) and CXCL12 further purified by affinity chromatography (Ni-NTA agarose resin) and Grace^®^ Vydac^®^ C18 reverse-phase HPLC column (Avantor, Søborg, Denmark). The purified CXCL12 was buffer exchanged to 20 mM acetate pH 4, concentrated and stored at −80 °C.

### 2.3. Expression, Purification and Biotinylation of Membrane Scaffold Proteins

Membrane Scaffold Protein (MSP) 1E3D1 expression and purification was carried out as previously described [45] but with adjusted storage buffer. Briefly, His-tagged MSP1E3D1 was expressed from the pET28a vector in *E. coli* BL21(DE3)pLysS (Agilent, Glostrup, Denmark). Ni-NTA agarose resin (Qiagen, København, Denmark) was used to purify MSP1E3D1 from cell lysate. The eluted protein was dialyzed against the storage buffer (20 mM HEPES pH 7.5, 150 mM NaCl and 5 mM sodium cholate), concentrated and stored at −80 °C. For biotinylation, MSP1E3D1 was incubated with a four-fold excess of EZ-Link NHS-PEG4-Biotin (Thermo Fisher Scientific, Roskilde, Denmark) for 1 h at room temperature (RT). The reaction was quenched by the addition of 5 mM TRIS pH 7.5. Biotinylation efficiency was assessed by pull-down of biotinylated MSP1E3D1 with Pierce High Capacity Streptavidin Agarose beads (Thermo Fisher Scientific, Roskilde, Denmark), and subsequent SDS-PAGE analysis.

### 2.4. Reconstitution of ACKR3 into Nanodiscs

In total, 16:0–18:1 1-palmitoyl-2-oleoyl-glycero-3-phosphocholine (POPC; Merck Life Science, Søborg, Denmark) lipids dissolved in chloroform were dried using nitrogen gas. After complete evaporation of chloroform, the lipid film was solubilized in cholate buffer pH 7.5 (50 mM HEPES pH 7.5, 150 mM NaCl, 200 mM sodium cholate) at a final concentration of 100 mM.

ACKR3 was reconstituted into nanodiscs (NDs) as previously described [44], with the following modifications. Briefly, purified ACKR3, MSP1E3D1 and POPC lipids were mixed in ND buffer (50 mM HEPES pH 7.5, 150 mM NaCl) to reach a final concentration of 2.85 µM, 29.85 µM and 3.14 mM, respectively. In parallel, a reaction mixture devoid of ACKR3 was prepared. To exceed the critical micelle concentration, cholate buffer was additionally added to both reaction mixtures to a final sodium cholate concentration of 26.3–27.1 mM. The reaction mixtures (final volume of 194 or 219 µL for reconstitutions with or without ACKR3, respectively) were incubated at 4 °C for 20 min with gentle stirring. Self-assembly of NDs was initiated by the addition of Bio-Beads SM-2 Resin (Bio-Rad, København, Denmark) and subsequent incubation overnight at 4 °C under continuous stirring. The beads were removed and the NDs purified by size-exclusion chromatography [SEC; ProteoSEC Dynamic 11/30 6-600 HR SEC Column (Bionordika, Herlev, Denmark)]. Note that the concentration of HEPES pH 7.5 in the ND buffer was doubled compared to the original protocol, and that the additional purification by Talon IMAC resin was omitted.

### 2.5. Nanodisc Analysis by Western Blot and SDS-PAGE

Western blot and SDS-PAGE analysis were used to quantify the ACKR3 and MSP1E3D1 concentration, respectively, and assess the purity of the pooled ND-containing SEC fractions. For the analysis, parts of the pooled fractions were concentrated using a 100 kDa molecular weight cutoff Amicon Ultra 0.5 centrifugal filter (Merck Life Science, Søborg, Denmark).

For Western blot analysis, concentrated NDs and various concentrations of detergent purified ACKR3 (0.01–0.50 µg) were run on a 4–15% Criterion TGX polyacrylamide gel (Bio-Rad, København, Denmark). After transfer to a LF PVDF membrane (Bio-Rad, København, Denmark) using the Trans-Blot Turbo Transfer System (Bio-Rad, København, Denmark), the membrane was blocked for 30 min in Intercept (TBS) blocking buffer (LI-COR Biotechnology, Bad Homburg, Germany). The membrane was transferred to buffer A [1:1 mixture of Intercept (TBS) blocking buffer, and PBS supplemented with 1 mM CaCl_2_ and 1 mM MgCl_2_] containing the mouse ANTI-FLAG M2 antibody (Merck Life Science, Søborg, Denmark) in a 1:2000 dilution. After an overnight incubation with gentle shaking at 4 °C, the primary antibody was removed and the membrane washed three times with TBS-0.05% Tween-20. After the last washing step, the membrane was incubated for 1 h with IRDye 800 CW goat anti-mouse IgG secondary antibody (LI-COR Biotechnology, Bad Homburg, Germany) diluted 1:15,000 in buffer B [1:1 mixture of Intercept (TBS) blocking buffer and TBS-0.05% Tween-20]. The membrane was washed twice with TBS-0.05% Tween-20 and once with PBS supplemented with 1 mM CaCl_2_ and 1 mM MgCl_2_. All washing steps were performed at RT and for 10 min under gentle shaking. The membrane was imaged using a LI-COR scanner and the relative band intensities were analyzed with ImageJ (version 1.52a, National Institutes of Health, Bethesda, MD, United States) [46].

For SDS-PAGE analysis, concentrated and unconcentrated NDs, and a dilution series of MSP1E3D1 (0.06–1.00 µg) were run on a 4–15% Criterion TGX polyacrylamide gel (Bio-Rad, København, Denmark). The relative band intensities of the Coomassie-stained gel were analyzed with ImageJ (version 1.52a, National Institutes of Health, Bethesda, MD, United States) [46].

### 2.6. Scintillation Proximity Assay

The SPA experiments were conducted in a total volume of 100 µL assay buffer containing 60 mM HEPES pH 7.5, 200 mM NaCl, 3.98% (*v*/*v*) glycerol, 0.04% (*w*/*v*) bovine serum albumin (BSA) or as specified in Table 1. [^125^I]-CXCL12 (Perkin Elmer, Ballerup, Denmark) and CXCL12 were prepared in 60 mM HEPES pH 7.5, 120 mM NaCl and 0.2% (*w*/*v*) BSA.

For kinetic experiments, ACKR3 NDs (180–200 fmol ACKR3, or 17–679 fmol ACKR3 for biotin–beads ratio optimization) or corresponding amount of empty NDs were mixed with streptavidin-coated polyvinyltoluene SPA beads (96–107 µg in a ratio of 30 µg of beads per pmol biotin, or 90 µg for biotin–beads ratio optimization; Perkin Elmer) in a white 96-well low-binding surface OptiplateTM (Perkin Elmer, Ballerup, Denmark) and a total reaction volume of 89 (dissociation experiments) or 90 µL (association experiments). The plate was incubated for 30 min at RT under gentle shaking to immobilize the NDs onto the beads surface. The reaction mixture was quickly mixed with 10 µL [^125^I]-CXCL12 at a final concentration of 60 pM and the association continuously monitored on a TopCount NXT (Perkin Elmer) in 1 min reads at 20 °C. For dissociation experiments, association was followed until an equilibrium was reached and dissociation subsequently initiated by the addition of 1 µL of 10 mM small molecule agonist VUF11207 (Merck Life Science, Søborg, Denmark; [43]) in assay buffer. Dissociation of [^125^I]-CXCL12 was measured as described for association experiments. Competition binding experiments were conducted as the association experiments but with various concentrations of unlabeled CXCL12 or ACKR3 agonist TC14012 (Tocris Bioscience, Abingdon, UK; [47]), which were added prior to the 30 min incubation. In addition, the reading time was increased to 3 min. Incubation times exceeding the recommendations of three to five dissociation half-lives (*T*_1/2_ = 0.693/*k*_off_) were used to sufficiently approach equilibrium [27,30,31].

### 2.7. Data Analysis

Specific binding was calculated from averaged duplicates by subtracting NSB to empty NDs from total binding to ACKR3 NDs. Association and dissociation experiments were fitted to a one-phase association or one-phase exponential decay model, respectively, using GraphPad Prism version 9.3.0 (GraphPad Software, Inc., San Diego, CA, USA). CXCL12 binding to ACKR3 is a protein–protein interaction [19], and has been previously shown to follow pseudo-first-order kinetics with respect to receptor concentration when the receptor is present at a concentration far exceeding that of the chemokine [48]. Since the total ACKR3 concentration [*R*_tot_] was >30-fold the concentration of [^125^I]-CXCL12, the *k*_on_ values were calculated from the fitted *k*_on(obs)_ and averaged *k*_off_ according to Equation (1). The dependence of *k*_on(obs)_ on the ACKR3 but not on the [^125^I]-CXCL12 concentration was experimentally confirmed (Figure S1).

(1) $$k_{\mathrm{on}}=\frac{k_{\mathrm{on}(\mathrm{obs})}-{\;k}_{\mathrm{off}}}{{[R}_{\mathrm{tot}}]\;}$$

*K*_d_ was calculated from

(2) $$K_{d}=\frac{k_{\mathrm{off}}}{k_{\mathrm{on}}\;}$$

using averaged *k*_off_ and *k*_on_ values. IC_50_ values were obtained by fitting the competition binding data to a one-site fit logIC_50_ model in GraphPad Prism. As the extrinsic IC_50_ values strongly depend on, e.g., substrate concentration [49,50], the intrinsic *K*_i_ values were used instead for data comparisons. Given the high receptor concentration relative to the radioligand concentration, IC_50_ values do not correctly reflect *K*_i_ values due to radioligand depletion [51]. To determine *K*_i_ values, the Goldstein–Barrett correction [52] was therefore applied following Equations (3)–(5):

(3) $$K_{i}=\frac{{\mathrm{IC}}_{50}}{{1+[L}_{50}{]/K}_{d}+2\times {([L}_{50}]\;-{\;[L}_{0}{])/[L}_{0}]\;}$$

(4) $$L_{0}=\frac{{[L}_{\mathrm{tot}}]}{{1+[R}_{\mathrm{tot}}{]/[K}_{d}]\;}$$

(5) $$L_{50}{=[L}_{\mathrm{tot}}]\;-\frac{{[L}_{\mathrm{tot}}]-{[L}_{0}]}{2}$$

where [*L*_tot_] is the [^125^I]-CXCL12 concentration. All values provided in the text or tables are mean ± standard error of the mean (SEM) if not stated otherwise.

## 3. Results

### 3.1. Principle of the Assay

**Figure 1:**
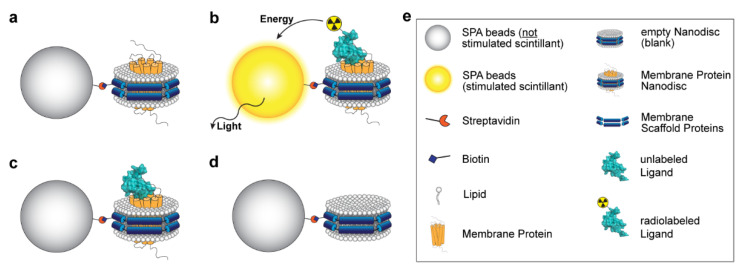
Principle of the Scintillation Proximity Assay applied to nanodiscs (NDs). (**a**) The membrane protein of interest is reconstituted into biotinylated NDs. (**b**) The high affinity of biotin to streptavidin allows the binding of the NDs to streptavidin-coated SPA beads. These beads contain a scintillator that converts the energy of a radioactive decay to light. Consequently, if a radioligand binds to the immobilized membrane protein, the released energy is transferred to the scintillator and light is generated. (**c**) In the presence of an unlabeled ligand, the two ligands compete for binding to the membrane protein. If the unlabeled ligand outcompetes the radioligand, the light emission ends because the released energy of the radioligand is too distant from the scintillator. Light measurements therefore directly provide information on ligand–receptor binding. (**d**) Empty NDs serve as a control for non-specific binding. (**e**) Assay components. The depicted ligand is CXCL12 (PDB: 1A15 [54]).

The principle of the SPA (Figure 1) is the conversion of energy into light by a scintillator [53]. Here, the scintillator is contained in streptavidin-coated SPA beads. Biotinylated NDs were immobilized onto the beads’ surface by streptavidin-biotin binding. In the case of receptor-containing NDs (Figure 1a), radiolabeled ligand binds to the receptor bringing the radioisotope and scintillator in close proximity to each other (Figure 1b). As a result, the energy released by the radioisotope decay is transferred to the scintillator. The subsequent light emission is measured as a direct readout of receptor–ligand binding. In the case of unlabeled ligands, no light is generated by the scintillators (Figure 1c). Subtraction of the binding to empty NDs (Figure 1d), i.e., NSB, from the one to receptor-containing NDs yields the specific binding.

### 3.2. Sample Preparation

**Figure 2:**
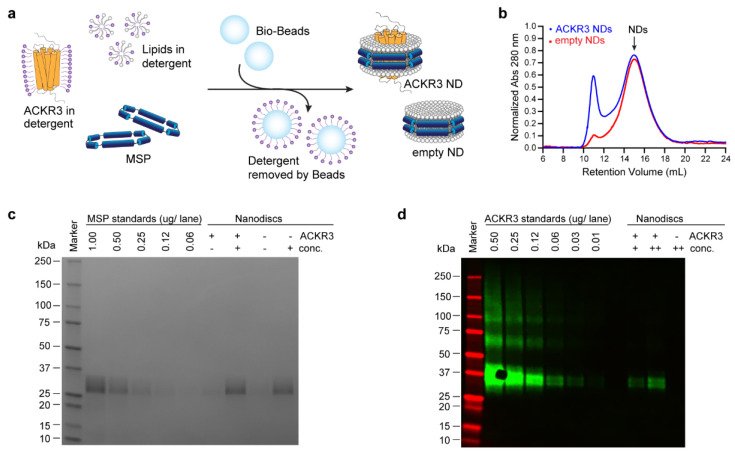
Nanodisc (ND) assembly and analysis. (**a**) ND reconstitution. ACKR3, amphipathic Membrane Scaffold Proteins (MSP) and lipids of choice are mixed. Subsequent addition of Bio-Beads removes the detergent and initiates the formation of NDs. (**b**) Size-exclusion chromatography trace of empty (red) and ACKR3 (blue) NDs. ACKR3 NDs generated a bigger aggregation peak compared to empty NDs. (**c**) Quantification of MSP by SDS-PAGE analysis. + and − refer to concentrated and unconcentrated ND fractions, respectively. (**d**) Quantification of ACKR3 by Western blot analysis. Various concentrations of ACKR3 solubilized in detergent (0.01–0.50 µg) or reconstituted into NDs (++ and +) are shown.

NDs allow the study of membrane proteins in a native-like environment while having full control over the composition of the lipid bilayer [55]. Previous studies showed that ACKR3 is stable [44] and remains fully functional [48] when reconstituted into NDs. The reconstitution of ACKR3-containing NDs is described in Figure 2a. In brief, Bio-Beads were added to a mixture of ACKR3, lipids and MSP, which removed the detergent and initiated the self-assembly of NDs. Aggregates were removed by SEC (Figure 2b). The collected ND fractions were pooled and analyzed by Western blot and SDS-PAGE (Figure 2c,d). In the Western blot, we included a dilution series of ACKR3 solubilized in detergent, which allowed the accurate quantification of ACKR3 in NDs (Figure 2d). For MSP quantification, we run SDS-PAGE with MSP standards (Figure 2c). Information on the MSP concentrations is important for a proper determination of the optimal ratio of NDs per SPA beads (i.e., streptavidin-biotin), which is discussed further in the next section. Moreover, knowing the MSP concentration of empty and receptor-containing ND samples is crucial for the appropriate usage of empty NDs as blanks for receptor-containing NDs.

### 3.3. Determination of the Optimal Biotin–Beads Ratio

**Figure 3:**
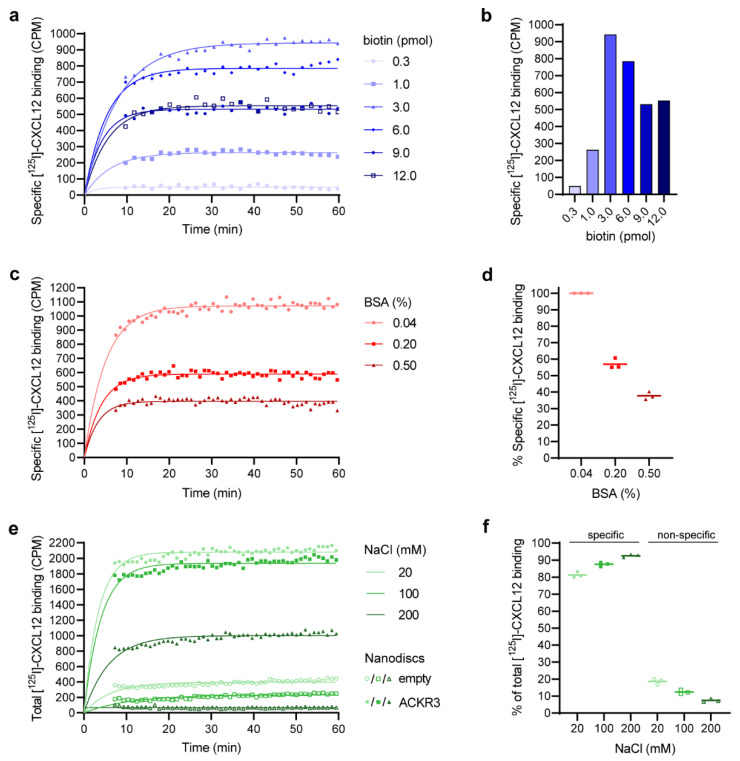
Optimization of the Scintillation Proximity Assay. (**a**,**b**) Determination of an optimal biotin–beads ratio of 30 µg beads per pmol biotin. In total, 90 µg SPA beads were incubated with different amounts of biotinylated nanodiscs (NDs). Radioligand was added and the signal measured for 60 min. The experiment was performed in duplicates and averaged. Blank subtraction yielded the specific binding. A one-phase association model was fit to the curves using GraphPad Prism version 9.2.0 (GraphPad Software, Inc, San Diego, CA, USA) and their plateaus plotted. (**c**,**d**) The reduction in Bovine Serum Albumin (BSA) in the system results in an increased amount of specific binding. Three independent experiments were performed in duplicates, and the specific binding determined and analyzed as in (**a**). One of the three replicates is depicted in (**c**), while (**d**) shows the data that were normalized to the signal at 0.04% BSA. (**e**,**f**) NaCl reduces non-specific binding. Three independent experiments were performed in duplicates with one of them given in (**e**). The total binding to empty (open symbols) and ACKR3 (solid symbols) NDs is shown at 20 mM (light green circles), 100 mM (green squares) and 200 mM (dark green triangles) NaCl. A one-phase association model was fit to the curves using GraphPad Prism. The percentages of non-specific and specific binding at equilibrium were calculated for each buffer condition and summarized in (**f**). Horizontal bars in (**d**,**f**) represent means. Based on the NaCl and BSA screen, 200 mM NaCl and 0.04% (*w*/*v*) BSA were included in the final optimized assay buffer.

In order to obtain a maximum assay window, i.e., signal-to-noise ratio, we optimized the biotin–beads ratio. To this end, we determined the specific binding of [^125^I]-CXCL12 to ACKR3 at varying concentrations of NDs (i.e., biotin) but constant amount of SPA beads (i.e., streptavidin). The found optimal biotin–beads ratio, given by a maximum amount of specific signal, is 30 µg of beads per pmol biotin (Figure 3a,b). While this number is in agreement with a previous study using biotinylated oligonucleotides [56], it is approximately three times higher than the one provided by the manufacturer (Perkin Elmer) or other studies [57,58]. Such a discrepancy between theoretical and actual beads’ binding capacity could be a result of steric hindrance [56]. Furthermore, note that there are two biotins per ND at a 100% biotinylation efficiency of the MSP. Overall, this emphasizes the need to examine the beads’ binding capacity specific for the system under investigation.

### 3.4. Reduction in Non-Specific Binding

To improve the sensitivity and specificity of the assay, we sought to minimize NSB by means of BSA. However, the specific signal decreased with increasing BSA concentrations (Figure 3c,d). For example, the specific signal dropped by approximately 20% and became insufficient after increasing the BSA concentration from 0.2% to 0.5% (*w*/*v*). Nevertheless, a complete removal of BSA was unfeasible given the “stickiness” of chemokines and their corresponding inherent NSB [59]. We addressed this trade-off between specific signal and NSB of the chemokine by using different buffers for the chemokine preparation and final reaction mixture (see Materials and Methods). In brief, the chemokine was prepared in buffer containing 0.2% (*w*/*v*) BSA. Its subsequent addition to the reaction mixture resulted in a final BSA concentration of 0.04% (*w*/*v*). These measures allowed the gain of a specific signal of about 60% compared to 0.5% (*w*/*v*) BSA (Figure 3c,d) as well as a reduction in chemokine loss. Nonetheless, an alternative strategy was required to reduce the NSB in the assay. We therefore optimized the NaCl concentration (Figure 3e,f), which enabled on average about a 5-fold reduction in NSB. Moreover, the blank signal remained constant over time and was on average around 7% of the receptor signal. All of this contributes to improved data accuracy and reliability.

### 3.5. Assay Validation: Binding Kinetic Studies on the CXCL12–ACKR3 Interaction

**Figure 4:**
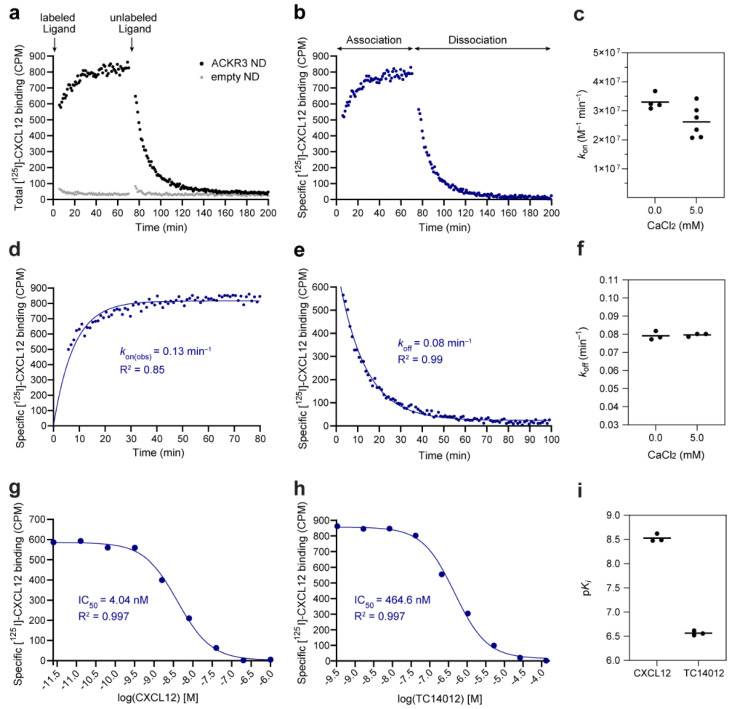
Determination of the kinetic rate constants and equilibrium binding metrics of the ACKR3–CXCL12 interaction by means of the Scintillation Proximity Assay (SPA). (**a**) Representative association and dissociation of the [^125^I]-CXCL12/ACKR3 complex monitored by SPA using empty (grey) and ACKR3-containing (black) nanodiscs (NDs). Black arrows indicate the initiation of [^125^I]-CXCL12 association or dissociation by the addition of either [^125^I]-CXCL12 or unlabeled ACKR3 agonist VUF11207, respectively. (**b**) Specific binding of (**a**) calculated by subtracting the non-specific binding to empty NDs from the total binding to ACKR3 NDs. (**c**) Association rate constants (*k*_on_) of the [^125^I]-CXCL12/ACKR3 complex. (**d**,**e**) Representative association (**d**) and dissociation (**e**) curve obtaining *k*_on(obs)_ and *k*_off_, respectively. (**f**) Dissociation rate constants (*k*_off_) of the [^125^I]-CXCL12/ACKR3 complex. Kinetic data are from three to six independent experiments performed in duplicates, and in the presence or absence of CaCl_2_. *p* = 0.051 (*k*_on_), *p* = 0.751 (*k*_off_), unpaired two-tailed t test. (**g**,**h**) Homologous (**g**) and heterologous (**h**) competition of unlabeled CXCL12 and ACKR3 agonist TC14012, respectively, with [^125^I]-CXCL12 for ACKR3 binding. Competition binding data were well fit by the models with R^2^ > 0.99 in all cases. (**i**) p*K*_i_ values calculated from IC_50_ values of three independent experiments performed in duplicates. Data points of all representative curves display averaged duplicates. Horizontal bars in (**c**,**f**,**i**) represent means.

Next, we characterized the kinetics of CXCL12 binding to ACKR3 using the optimized SPA (Figure 3 and Table 1) to validate its suitability for kinetic profiling of chemokine–chemokine receptor interactions. Additionally, we evaluated the assay performance in the presence of divalent cations, as some of the 7TM receptors specifically require them for their physiological function [60] and they are common buffer additives in CXCL12–ACKR3 binding studies. Figure 4a,b show the association and dissociation of the [^125^I]-CXCL12/ACKR3 complex. The association was monophasic, reaching a plateau within approximately 30 min (Figure 4d). The dissociation was also monophasic, and both buffer conditions yielded nearly identical dissociation rate constants with *k*_off_ = 0.079 ± 0.001 min^−1^ (0 mM CaCl_2_) and *k*_off_ = 0.080 ± 0.001 min^−1^ (5 mM CaCl_2_) (Figure 4e,f). All dissociation experiments were very well described by the model represented by high correlation coefficients (R^2^ ≥ 0.98 in all cases). R^2^ values were slightly lower for the association experiments due to the faster association and the loss of some data points in the early part of the curve. The association rate constants derived from the obtained *k*_off_ and *k*_on(obs)_ were (3.30 ± 0.13) × 10^7^ M^−1^ min^−1^ (0 mM CaCl_2_) and (2.62 ± 0.22) × 10^7^ M^−1^ min^−1^ (5 mM CaCl_2_) (Figure 4c). A summary of all kinetic parameters is provided in Table 2.

The kinetics of CXCL12 binding to ACKR3 were previously described using a flow cytometry-based assay in live *Sf9* cells [48]. For comparison reasons, these data were refitted to a monophasic model, yielding *k*_off_ = 0.008 ± 0.002 min^−1^ and *k*_on_ = (1.49 ± 0.23) × 10^7^ M^−1^ min^−1^. The resulting *k*_on_ is in good accordance with the SPA data while our *k*_off_ is approximately 10-fold faster. However, it is difficult to directly compare the kinetic data of different assays that were not only run in different systems (NDs vs. *Sf9* cells) but also at different temperatures (temperature-controlled SPA at 20 °C vs. flow cytometry measurements at RT without temperature control) and in distinct buffer conditions. A major advantage of the described SPA compared to the flow cytometry measurements is that the SPA is a continuous assay. This means that the reactions are continuously monitored, allowing the collection of a larger number of data points revealing progress curves and potential artefacts. Moreover, it reduces noise and inaccuracies introduced by, e.g., sample preparation, handling and timing. Altogether, this results in generally improved data quality [61,62]. Importantly, calculated *K*_d_ values of 2.40 ± 0.10 nM (mean ± propagated error; 0 mM CaCl_2_) and 3.04 ± 0.26 nM (5 mM CaCl_2_) are in line with previously reported affinities ranging from 0.2 to 28 nM (median: 0.515 nM) [43,63,64,65]. All in all, we conclude that the presented SPA is a suitable tool for kinetic studies of chemokine–chemokine receptor interactions. Furthermore, the CaCl_2_ studies indicate that the SPA allows flexibility regarding the inclusion of additional divalent ions, and consequently the study of ion-dependent receptors.

### 3.6. Alternative Assay Application: Competition Binding Studies

The application of the SPA can additionally be expanded to competition binding experiments for the assessment of ligand potency (IC_50_) and affinity (*K*_i_). In the homologous competition of unlabeled CXCL12 with [^125^I]-CXCL12 for ACKR3 binding, an IC_50_ of 5.05 ± 0.51 nM was obtained (Figure 4g and Table 2). The corresponding *K*_i_ of 3.01 ± 0.30 nM is in good agreement with the *K*_d_ calculated from our kinetic rate constants (3.04 ± 0.26 nM) and the literature (0.2 to 28 nM, median: 0.515 nM [43,63,64,65]). Of note is the considerable inter-experimental variability in studies only providing IC_50_ values (0.014 to 5.9 nM [66,67,68,69,70,71,72]). In addition to the homologous competition binding experiment, we characterized the heterologous competition between [^125^I]-CXCL12 and TC14012 [47] for ACKR3 binding. TC14012 [47] is an ACKR3 cyclic peptide agonist that binds to the major orthosteric pocket of ACKR3 formed by transmembrane helix 3 to 7 [73]. The determined IC_50_ of 0.459 ± 0.029 µM was converted to a *K*_i_ of 0.274 ± 0.017 µM (Figure 4h,i and Table 2), which closely resembles the previously reported *K*_i_ of 0.157 µM [47]. Overall, the presented case studies on heterologous and homologous competition binding confirm that the SPA can also be applied for IC_50_ and *K*_i_ determination, which makes the SPA a versatile tool for the study of receptor–ligand interactions.

## 4. Discussion

In this work, we describe the application, optimization and validation of a scintillation proximity assay for real-time kinetic profiling of chemokine–chemokine receptor interactions using the example of the atypical chemokine receptor ACKR3 and its natural ligand CXCL12. In the presented homogenous SPA [53], the receptor is reconstituted into NDs and radioligand binding continuously monitored by scintillation light in real time. This enables the collection of large data sets that contain information on the progress of reactions as well as potential artefacts. The limited processing of the samples contributes to improved data accuracy [61,62]. By simultaneously measuring empty and receptor containing NDs, NSB is directly detected and accounted for in the data analysis. The illustrated case study revealed low NSB and a large assay window under optimized assay conditions, which further contributes to data accuracy and reliability. A benefit of the assay is the ability to directly determine *K*_d_ values as opposed to indirect measurements from competition binding data. However, a drawback is the need for a radioligand to follow direct binding to the receptor. Alternatively, the assay could be adapted to measure competition association experiments [74], which would enable indirect measurements of binding kinetics of unlabeled ligands.

An important feature of the assay is the usage of NDs. NDs allow the detergent-free study of receptor–ligand interactions, which is particularly essential for membrane proteins with impaired function and stability in the presence of a detergent [75]. Furthermore, the receptor oligomerization state as well as the composition and size of the lipid bilayer can be controlled [55]. Importantly, no modifications of the receptors are required [21] and both, the intra- and extracellular domain of the embedded receptor can be studied [55]. While NDs cannot fully capture the complexity of the lipid bilayer of living cells (e.g., membrane dynamics, lipid and bilayer asymmetry, great diversity of lipids, etc.) [76], they create an opportunity to investigate the effect of bilayer composition and properties on membrane protein structure and function. Although 7TM receptors and other membrane proteins were previously shown to be modulated by specific lipids [77,78], this field is still largely unexplored. Of particular interest to chemokine receptors could also be the re-introduction of glycosaminoglycans (GAGs) in NDs. GAGs are polysaccharides presented on cell surfaces and in extracellular matrices, which interact with chemokines and thereby affect chemokine localization and activity [79,80,81].

All in all, the combination of the mentioned benefits of NDs and the SPA principle makes the assay a powerful and versatile tool for the study of receptor–ligand interactions. Using the example of ACKR3, we demonstrate that the application of the SPA for kinetic and competition binding studies yields high quality data. Based on all these findings, we propose the introduced SPA as a promising tool to increase our kinetic understanding of chemokine–chemokine receptor interactions. This could aid future drug-discovery campaigns and shed light on the complexity of the chemokine–chemokine receptor network. Moreover, the presented SPA could provide a platform for the study of the interplay between membrane composition and chemokine receptor function.

## Figures and Tables

**Table 1:** Summary of experiment-specific Scintillation Proximity Assay (SPA) buffer adjustments. NSB, non-specific binding.

SPA Experiment	Buffer Adjustment *
NSB: BSA Screen	0.04–0.50% (*w*/*v*) BSA
NSB: NaCl Screen	20–200 mM NaCl
Kinetic measurements	0 or 5 mM CaCl_2_
Competition binding	5 mM CaCl_2_

* The standard assay buffer condition was 60 mM HEPES pH 7.5, 200 mM NaCl, 3.98% (*v*/*v*) glycerol and 0.04% (*w*/*v*) BSA.

**Table 2:** Kinetic and equilibrium binding metrics of ligand binding to ACKR3 obtained from the Scintillation Proximity Assay. Values represent mean ± standard error of the mean (SEM) of three to six independent experiments performed in duplicates. Overall, 0 and 5 mM CaCl_2_ refer to experiment performance in the absence or presence of CaCl_2_, respectively. CI, confidence interval.

	0 mM CaCl_2_			5 mM CaCl_2_	
	Mean ± SEM	95% CI		Mean ± SEM	95% CI
***k*_on_** (**M^−1^ min^−1^**)	(3.30 ± 0.13) × 10^7^	(3.04 to 3.55) × 10^7^		(2.62 ± 0.22) × 10^7^	(2.19 ± 3.06) × 10^7^
***k*_off_** (**min^−1^**)	0.079 ± 0.001	0.076 to 0.082		0.080 ± 0.001	0.079 to 0.081
***T*_1/2_** (**min**)	8.76 ± 0.15	8.47 to 9.06		8.70 ± 0.06	8.59 to 8.81
***K*_d_** (**nM**) *	2.40 ± 0.10	-		3.04 ± 0.26	-
**IC_50_** (**nM**)					
**CXCL12**	-	-		5.05 ± 0.51	4.06 to 6.04
**TC14012**	-	-		458.6 ± 29.0	401.8 to 515.4
***K*_i_** (**nM**)					
**CXCL12**	-	-		3.01 ± 0.30	2.42 to 3.60
**TC14012**	-	-		273.9 ± 17.3	240.0 to 307.9

* *K*_d_ values were calculated from averaged *k*_on_ and *k*_off_, with errors propagated from their respective SEM.

## Data Availability

Source data are provided with this paper.

## Supplementary Materials

The following supporting information can be downloaded at: https://www.mdpi.com/article/10.3390/cells11081317/s1, Figure S1: The observed association rate constant *k*_on(obs)_ depends on the ACKR3 concentration but not on the [^125^I]-CXCL12 concentration.
