# Supplementary material for: A Scintillation Proximity Assay for Real-Time Kinetic Analysis of Chemokine–Chemokine Receptor Interactions
Source: Cells. 2022 Apr 13;11(8):1317. doi: 10.3390/cells11081317 (PMC9024993; doi:10.3390/cells11081317)
Supplement: Supplementary file 1 [file cells-11-01317-s001.zip › cells-1664869-SI.pdf]

Supplementary

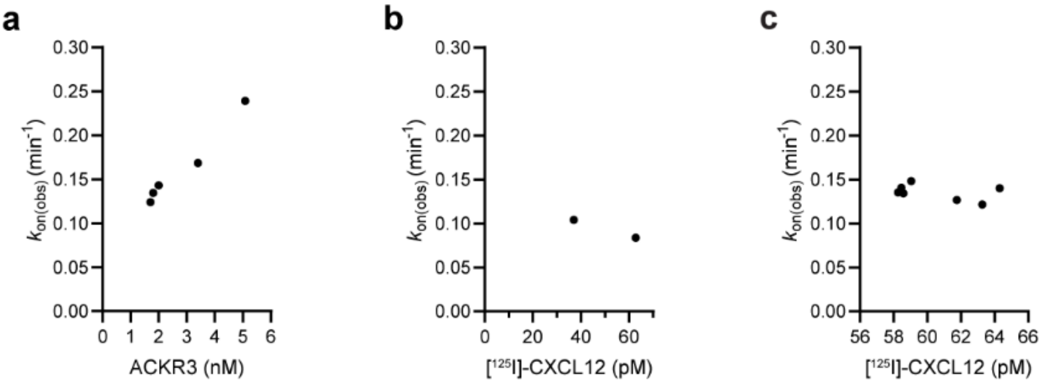

Figure S1. The observed association rate constant $k_{on(obs)}$ depends on the ACKR3 concentration but not on the [^{125}I]-CXCL12 concentration. (**a**) $k_{on(obs)}$ increases with increasing ACKR3 concentration. $k_{on(obs)}$ were determined at various ACKR3 concentrations but constant [^{125}I]-CXCL12 concentration (~61 pM); (**b**,**c**) $k_{on(obs)}$ and the [^{125}I]-CXCL12 concentration are uncorrelated. Different [^{125}I]-CXCL12 concentrations were tested at 1.6 nM (**b**) and 2.0 nM (**c**) ACKR3. All experiments were performed in duplicates. Subtraction of the non-specific binding to empty nanodiscs (NDs) from the total binding to receptor containing NDs yielded the specific binding. One-phase association models were fitted to the specific binding curves using GraphPad Prism version 9.3.0 (GraphPad Software, Inc., San Diego, CA) to obtain $k_{on(obs)}$. Different buffer conditions were used for the tests as follows: (**a**) 200 mM NaCl, 3.98% (*v*/*v*) glycerol, 60 mM HEPES pH 7.5, 0.04% (*w*/*v*) BSA; (**b**) 213 mM NaCl, 3.98% (*v*/*v*) glycerol, 66 mM HEPES pH 7.5; (**c**) as (**a**) but with 5 mM $CaCl_2$.
